# Anomalies of the right hepatic artery in periampullary cancer treatment: are pathological and clinical outcomes different? A single tertiary referral center retrospective analysis

**DOI:** 10.1007/s00423-024-03263-6

**Published:** 2024-02-23

**Authors:** Davide De Sio, Chiara Lucinato, Edoardo Panza, Giuseppe Quero, Vito Laterza, Carlo Alberto Schena, Claudio Fiorillo, Flavia Taglioni, Roberta Menghi, Fabio Longo, Fausto Rosa, Antonio Pio Tortorelli, Vincenzo Tondolo, Sergio Alfieri

**Affiliations:** 1 Pancreatic Surgery Unit, Department of Surgery, Gemelli Pancreatic Center, CRMPG (Advanced Pancreatic Research Center), Fondazione Policlinico Universitario “Agostino Gemelli” IRCCS, Largo Agostino Gemelli, 8, 00168 Rome, Italy; 2 Università Cattolica del Sacro Cuore Di Roma, Largo Francesco Vito 1, 00168 Rome, Italy; 3 General Surgery Unit, Fatebenefratelli Isola Tiberina – Gemelli Isola, Via di Ponte Quattro Capi, 39, 00186 Rome, Italy

**Keywords:** Hepatic artery, Pancreatic cancer, Pancreatic surgery, Periampullary cancer, Pancreatoduodenectomy

## Abstract

**Purpose:**

Anomalies of the right hepatic artery (RHA) may represent an additional challenge in pancreatoduodenectomy (PD). The aim of this study is to assess the potential impact of variations in hepatic arterial anatomy on perioperative outcomes.

**Methods:**

PDs performed for periampullary malignancies between 2017 and 2022 were retrospectively enrolled and subdivided in two groups: modal pattern of vascularization (MPV) and anomalous pattern of vascularization (APV). A propensity score matching (PSM) analysis was conducted to homogenize the two study populations. The two groups were then compared in terms of perioperative outcomes and pathological findings.

**Results:**

Thirty-eight patients (16.3%) out of 232 presented a vascular anomaly: an accessory RHA in 7 cases (3%), a replaced RHA in 26 cases (11.2%), and a replaced HA in 5 cases (2.1%). After PSM, 76 MPV patients were compared to the 38 APV patients. The incidence rate of postoperative complications was comparable between the two study populations (*p*=0.2). Similarly, no difference was detected in terms of histopathological data, including margin status. No difference was noted in terms of intraoperative hemorrhage and vascular resection.

**Conclusion:**

When PDs are performed in high-volume centers, the presence of an APV of the RHA does not relate to a significant impact on perioperative complications. Moreover, no influence was noted on histopathological findings.

## Introduction

Pancreatoduodenectomy (PD) is the gold standard of treatment of periampullary malignancies and is widely known as a complex surgical procedure, burdened by a rate of postoperative complications up to 60% and a related mortality rate over 5% in low-volume centers [1]. In this context, the high variability of the regional vascular anatomy may increase the surgical technical difficulty. Notably, anomalies of the hepatic arteries are encountered in the 25–45% of cases, and variations of the right hepatic artery (RHA) represent the majority of them (12–18% of cases) [2, 3]. This has brought several authors to analyze the incidence rate of such variants and propose a classification with the aim of providing a common nomenclature. Currently, the most widely used are those proposed by Michels et al. [2] and Hiatt et al. [3], although a more comprehensive and complex classification has been more recently introduced by Yan et al. [4]. On the counterpart, contrasting results are present in the literature on the potential impact of on an aberrant RHA on short- and long-term outcomes of patients undergoing PD. Traverso advocated a potentially higher risk of biliary fistula (BF), possibly due to ischemia of the common bile duct stump following RHA skeletonization [5]. Furthermore, some authors reported an increased risk of R1 resections and local recurrence in case of anomalies of the RHA [6, 7]. Conversely, other authors did not evidence any difference when comparing patients with modal vascularization with those with anomalies of the RHA [8–10].

These conflicting results may find justification in the poor quality of data analyzed, mainly derived from small and non-homogeneous populations, as well as from non-referral centers for the surgical treatment of pancreatic diseases.

Based on these premises, the aim of this study was to assess the impact of anomalies of the RHA on perioperative outcomes after PD in a high-volume tertiary referral center for the surgical treatment of pancreatic diseases. In order to accomplish this purpose, patients who underwent PD for periampullary carcinomas with and without an aberrant RHA were compared. In addition, a propensity score matching (PSM) analysis was conducted to homogenize the two study populations.

## Material and methods

### Patient selection and data collection

After Institution Review Board approval, all patients who underwent PD at the Pancreatic Surgery Unit of the Fondazione Policlinico “Agostino Gemelli” IRCCS of Rome for a periampullary carcinoma from 2017 to 2022 were retrospectively enrolled in the study. All cases were preoperatively discussed at the multidisciplinary meeting where an expert radiologist assessed neoplasms resectability and the presence of vascular anomalies based on of the whole-body computed tomography (CT) scan performed preoperatively. According to the documented vasculature, the study population was subdivided into patients with a modal pattern of vascularization (MPV group) and patients with an anomalous pattern of vascularization (APV group). When detected, the variant of the RHA originating from the SMA was classified as accessory (aRHA) or replaced (rRHA), according to the definition by Michels et al. [2] Moreover, the presence of a common or proper hepatic artery entirely originating from the SMA was also recorded (rHA). Specifically, the common rHA was defined as the hepatic artery originating from the SMA that branches into the gastroduodenal artery and the proper hepatic artery (and subsequently into the right and left hepatic arteries), while the proper rHA was defined as the hepatic artery originating from the SMA that branches into the right and left hepatic arteries. In this last case, the gastroduodenal artery originates directly from the celiac trunk.

The preoperative radiological evidence of tumoral infiltration of a rRHA and rHA was considered a contraindication to resection.

Perioperative data were retrospectively collected from prospectively maintained databases. Clinico-demographic characteristics included sex, age, body mass index (BMI), and American Society of Anesthesiologists score. The following perioperative features were also collected: tumor localization, preoperative biliary drainage, neoadjuvant treatment (NAT), operative time, estimated blood loss (EBL), associated vascular resection, postoperative complications, and length of hospital stay (LOS). Clavien–Dindo classification was used to classify postoperative complications [11]. Postoperative pancreatic fistula (POPF), delayed gastric emptying (DGE), and post-pancreatectomy hemorrhage (PPH) were defined and classified according to the International Study Group of Pancreatic Surgery criteria [12–14], while the International Study Group of Liver Surgery criteria were used to define and classify BF [15]. Postoperative mortality was also recorded and defined as any death occurring within 30 days from surgery.

The following histopathological data were additionally evaluated: tumor dimension and grading, number of harvested lymph nodes, number of positive lymph nodes, and margin status. Margin status was defined as R1 when tumoral cells were less than 1 mm from the resection margin [16]. The 8th edition of the AJCC/UICC system was used for TNM staging [17].

### Operative technique

As previously reported [18, 19], a PD with a child reconstruction was performed in all cases. Independently of the presence of a vascular variant, the operative approach and surgical technique did not vary. Specifically, after the division of the pancreatic head, a circumferential, down-to-up anticlockwise dissection of the retroperitoneal lamina was conducted from the portal/superior mesenteric vein (PV/SMV). After the ligation of the superior and inferior pancreatoduodenal veins, the PV/SMV was retracted to the left, and dissection was continued dorsally to it. During this phase, the identification of the aberrant vessel was obtained with the aid of palpation, and after its identification, dissection was carried out until its origin from the SMA and followed the SMA right circumference. For the reconstruction phase, the same bowel loop was used to create a duct-to-mucosa pancreaticojejunostomy, the hepaticojejunostomy, and the gastrojejunostomy. The gastro-jejunal anastomosis was performed at least 60 cm from the hepaticojejunostomy in a side-to-side, antecolic and antiperistaltic manner. In all cases, the specimen was inked along the retroportal lamina margin for histopathological evaluation.

### Study outcomes

The primary endpoint of the study was to compare the MPV and APV cohorts in terms of postoperative clinical outcomes. The secondary endpoint was a further comparison between the two study populations in terms of pathological findings, with a particular focus on margin status.

### Statistical analysis

Continuous data were reported as median and quartile rank (QR), while all categorical variables were expressed as number and percentages. Student’s *t* tests, Mann–Whitney *U* tests, Fisher’s tests, and χ^2^ test were used for the univariate analysis. A *p* value ≤ 0.05 was considered as statistically significant for all the analyses performed. A logistic regression model was performed for the PSM using the following covariates, deemed as potential confounding factors: age, sex, BMI, tumor location, and NAT. Patients were then matched with a 2:1 ratio using a caliper size of 0.2. All tests were performed using SPSS version 25 for Windows (SPSS Inc., Chicago, IL, USA).

## Results

**Fig. 1:**
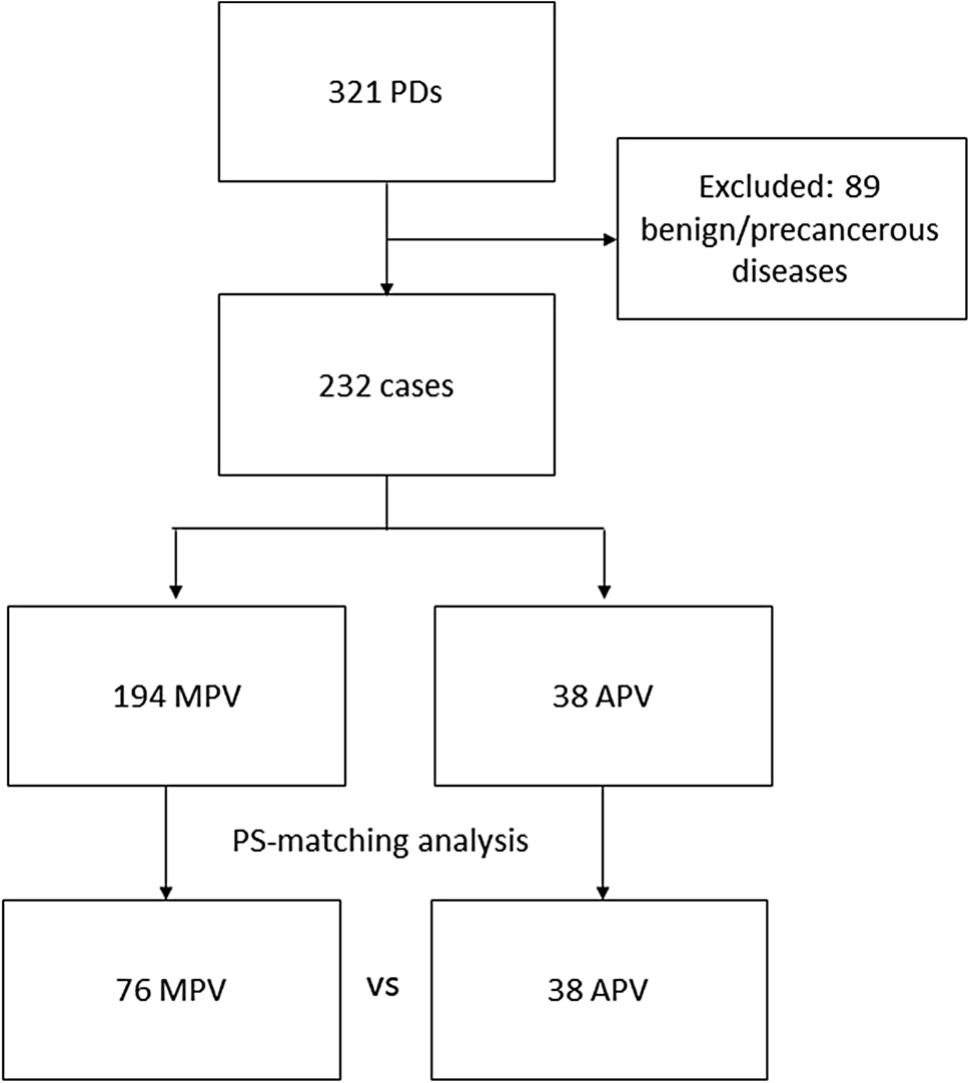
Flowchart of patients included in the study. PD, pancreatoduodenectomy; MPV, modal pattern of vascularization; APV, anomalous pattern of vascularization; PS, propensity score

**Fig. 2:**
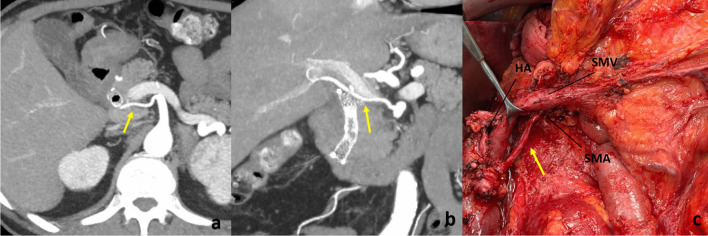
***a*** Axial view in CT scan of an aRHA (yellow arrow). ***b*** Coronal view in CT scan of an aRHA. ***c*** Intraoperative view of an aRHA after the removal of the surgical specimen and before the reconstructive phase. aRHA, accessory right hepatic artery; HA, hepatic artery; SMV, superior mesenteric vein; SMA, superior mesenteric artery

From January 2017 to December 2022, 321 patients underwent PD at the Pancreatic Surgery Unit of the Fondazione Policlinico Universitario Agostino Gemelli IRCCS of Rome. Of these, 232 (72.3%) were diagnosed with a periampullary carcinoma and were therefore included in the analysis (Fig. 1). An APV was preoperatively detected at the CT scan in 38 patients (16.4%) and further confirmed intraoperatively (Fig. 2). The remaining patients presented a MPV.

After PSM, 76 patients with a MPV were compared to 38 patients with an APV. The presence of APV was classified as follows: 7 (18.4%) patients had an aRHA, 26 (68.4%) a rRHA, and 5 (13.2%) a rHA arising from the SMA. As a whole, NAT was performed in 10 (8.8%) patients. Sixty-eight patients presented severe jaundice at the diagnosis, and a biliary stent was preoperatively placed via ERCP in 63 of them (55.3%), while a trans-hepatic biliary drainage was positioned in 5 cases (4.4%). In terms of tumor location, 24 patients (21.1%) presented an ampullary cancer, 78 (68.4%) a pancreatic ductal adenocarcinoma of the head of the pancreas, 3 (2.6%) of the uncinate process, 5 (4.4%) had a distal bile duct adenocarcinoma, and 4 (3.5%) a duodenal adenocarcinoma. Clinico-demographic characteristics and their comparison between the two study cohorts are reported in Table 1.

**Table 1:** Clinico-demographic characteristics of the whole population and of the two study cohorts, before and after PSM

	Before PSM				After PSM		
	Whole population (*n*=232)	MPV cohort (*n*=194)	APV cohort (*n*=38)	*p*	MPV cohort (*n*=76)	APV cohort (*n*=38)	*p*
Sex, *n* (%)							
*Male*	125 (53.9)	107 (55.2)	18 (47.4)	0.37	42 (55.3)	18 (47.4)	0.43
*Female*	107 (46.1)	87 (44.8)	20 (52.6)		34 (44.7)	20 (52.6)	
Age (years), *median (QR)*		69 (62–76)	69 (57–75)	0.84	68 (59–74)	69 (59–75)	0.79
BMI, *n (%)*							
*18.5–24.9*	144 (62.1)	118 (60.8)	26 (68.4)	0.37	52 (68.4)	25 (65.8)	0.78
*25–30*	79 (34)	67 (34.6)	12 (31.6)		24 (31.6)	13 (34.2)	
*>30*	9 (3.9)	9 (4.6)					
ASA score, *n (%)*							
*I*	18 (7.8)	14 (7.2)	4 (10.5)	0.52	6 (7.9)	4 (10.5)	0.78
*II*	150 (64.6)	124 (63.9)	26 (68.4)		48 (63.2)	26 (68.4)	
*III*	64 (27.6)	56 (28.9)	8 (21.1)		22 (28.9)	8 (21.1)	
Preoperative diabetes, *n (%)*	51 (22)	42 (21.6)	9 (23.7)	0.78	14 (18.4)	9 (23.7)	0.51
Preoperative biliary drainage, *n (%)*	132 (56.9)	111 (57.3)	21 (55.3)	0.49	47 (61.8)	21 (55.3)	0.72
Tumor location, *n (%)*							
*Ampulla*	54 (23.3)	45 (23.2)	9 (23.7)	0.57	15 (19.7)	9 (23.7)	0.97
*Pancreatic head*	131 (56.5)	106 (54.7)	25 (65.8)		53 (69.7)	25 (65.8)	
*Pancreatic neck*	9 (3.9)	9 (4.6)					
*Uncinate process*	16 (6.8)	15 (7.7)	1 (2.6)		2 (2.6)	1 (2.6)	
*Distal bile duct*	13 (5.6)	11 (5.7)	2 (5.3)		3 (3.9)	2 (5.3)	
*Duodenum*	9 (3.9)	8 (4.1)	1 (2.6)		3 (3.9)	1 (2.6)	
Neoadjuvant therapy, *n (%)*	41 (17.8)	37 (19.1)	4 (10.5)	0.21	6 (7.9)	4 (10.5)	0.64
Type of anomaly, *n (%)*							
*aRHA*	7 (3)		7 (18.4)			7 (18.4)	
*rRHA*	26 (11.2)		26 (68.4)			26 (68.4)	
*rHA*	5 (2.1)		5 (13.2)			5 (13.2)	

*PSM* propensity score matching, *MPV* modal pattern of vascularization, *APV* anomalous pattern of vascularization, *BMI* body mass index, *ASA* American Society of Anesthesiologists, *aRHA* accessory right hepatic artery, *rRHA* replaced right hepatic artery, *rHA* replaced hepatic artery

### Perioperative outcomes

The median operative time was comparable between the two study groups (342 (300–386) vs 337 (316–390) min in the MPV and APV cohorts, respectively, *p* = 0.79). No arterial resection was performed in any case, and all the anomalous arteries were preserved. No injury of the aberrant arteries occurred intraoperatively. A venous resection was performed in 13 cases (11.4%): 3 (7.9%) in the APV group and 10 (13.2%) in the MVP group (*p* = 0.4). Comparable EBL values were documented in the two study cohorts (*p*=0.68). As a whole, 36 (31.6%) patients developed a Clavien–Dindo ≥3 postoperative complication without any difference between the two cohorts (*p* = 0.2). The incidence rate of a clinically relevant pancreatic fistula (B/C) was also comparable: 35.2% (24 cases) in the MPV group and 31.6% (12 cases) in the APV group (*p* = 0.31). PPH occurred in 6 patients (7.9%) of the MPV and 2 (5.3%) cases of the APV cohort (*p* = 0.6). As regards to BF, no difference was detected between the two study cohorts in terms of incidence rate (4 (5.3%) and 3 (7.9%) patients of the MPV and the APV cohorts, respectively (*p* = 0.58)). Moreover, no difference was detected in term of BF severity: 2 grade B (2.6%) and 2 grade C (2.6%) BFs in the MPV *vs* 3 grade B BFs in the APV cohort (*p* = 0.27).

Seventeen patients required a reoperation (14–18.4% in the MPV group and 3–7.9% in the APV cohort; *p* = 0.35), due to grade C POPF in 6 cases, BF in 1 case, PPH in 4 patients, dehiscence of the gastrojejunostomy in 3 cases, and bowel ischemia in 4 cases. Median LOS was 14 days in both groups (*p* = 0.61). Overall in-hospital mortality was 2.6% (3 patients), with no difference between the two cohorts (1 (2.6%) in the APV and 2 (2.6%) in the MPV groups, respectively; *p* = 1). Comparison of the intra- and postoperative outcomes is reported in Table 2.

**Table 2:** Perioperative outcomes of the two study cohorts

	MPV cohort (*n*=76)	APV cohort (*n*=38)	*p*
Operative time (min), *median (QR)*	342 (300–386)	337 (316–390)	0.79
EBL (ml), *median (QR)*	187 (90–210)	205 (95–285)	0.68
Vascular resection, *n (%)*	10 (13.2)	3 (7.9)	0.4
BF, *n (%)*	4 (5.3)	3 (7.9)	0.58
Grade of BF, *n (%)*			
*B*	2 (2.6)	3 (7.9)	0.27
*C*	2 (2.6)		
DGE, *n (%)*	24 (31.6)	8 (21.1)	0.24
Grade of DGE, *n (%)*			
*A*	6 (7.9)	3 (7.9)	0.28
*B*	7 (9.2)	4 (10.5)	
*C*	11 (14.5)	1 (2.6)	
POPF, *n (%)*	32 (42.1)	16 (42.1)	1
Grade of POPF, *n (%)*			
*BL*	8 (10.5)	4 (10.5)	0.31
*B*	18 (27.3)	12 (31.6)	
*C*	6 (7.9)		
PPH*, n (%)*	6 (7.9)	2 (5.3)	0.6
Clavien–Dindo ≥3, *n (%)*	27 (35.5)	9 (23.7)	0.2
Reoperation, *n (%)*	14 (18.4)	3 (7.9)	0.14
Mortality, *n (%)*	2 (2.6)	1 (2.6)	1
LOS (days), *median (QR)*	14 (10–22)	14 (11–20.5)	0.61

*MPV* modal pattern of vascularization, *APV* anomalous pattern of vascularization, *EBL* estimated blood loss, *BF* biliary fistula, *DGE* delayed gastric emptying, *POPF* postoperative pancreatic fistula, *BL* biochemical leak, *PPH* post-pancreatectomy hemorrhage, *LOS* length of hospital stay

### Histopathological findings (Table 3)

When considering pathological staging, the two study populations resulted comparable in terms of both T (*p* = 0.53) and N staging (*p* = 1). Furthermore, no difference was detected between the two groups in terms of tumor dimensions with a median of 25 (20.5–34) mm for the MPV and 26 (19.5–33.5) mm for the APV (*p* = 0.65). Histopathological evaluation documented an overall incidence of R1 of 26.3% (30 cases), with no difference between the MPV and the APV groups (22 (28.9%) and 8 (21.1%) respectively; *p* = 0.37). Furthermore, no difference was detected in terms of R1 location (*p* = 0.83). Tumor cells were found more frequently within 1 mm from the retroportal lamina margin, namely, in 12 cases (16%) in the MPV cohort and in 4 cases (10.5%) in the APV cohort. Other sites less frequently involved were the SMV groove in 7 (9.3%) and 3 (7.9%) patients of the MPV and APV cohorts, respectively, and the pancreatic resection margin in 1 case for each group (1.3% and 2.6% respectively). The median number of lymph nodes harvested was 22 in both cohorts (*p* = 0.76).

**Table 3:** Histopathological findings of the two study cohorts

	MPV cohort (*n*=76)	APV cohort (*n*=38)	*p*
Grading, *n (%)*			
*1*	2 (3)	3 (9.4)	0.34
*2*	49 (73.1)	24 (75)	
*3*	16 (23.9)	5 (15.6)	
T staging, *n (%)*			
*1*	14 (18.7)	9 (25.1)	0.53
*2*	44 (58.7)	21 (58.3)	
*3*	13 (17.3)	3 (8.3)	
*4*	4 (5.3)	3 (8.3)	
N staging, *n (%)*			
*0*	30 (39.5)	15 (39.5)	1
*1*	30 (39.5)	15 (39.5)	
*2*	16 (21.1)	8 (21)	
Tumor dimension (mm), *median (QR)*	25 (20.5–34)	26 (19.5–33.5)	0.65
Lymph nodes harvested, *median (QR)*	22 (16–28)	22 (18–27)	0.76
R1, *n (%)*	22 (28.9)	8 (21.1)	0.37
Site of R1, *n (%)*			
*Retroportal lamina*	12 (16)	4 (10.5)	0.83
*Pancreatic resection margin*	1 (1.3)	1 (2.6)	
*SMV groove*	7 (9.3)	3 (7.9)	
*Other*	1 (1.3)	-	

*MPV* modal pattern of vascularization, *APV* anomalous pattern of vascularization, *SMV* superior mesenteric vein

## Discussion

PD is widely known as one of the most challenging procedure in general surgery, whose complexity may be significantly increased by the concomitant presence of vascular anomalies of the periampullary region. Nevertheless, no unanimous data are currently present in the literature on the potential impact of vascular variants on perioperative outcomes, and scarce and contrasting evidences are currently present in particular on the potential role of an aberrant RHA.

According to our findings, the presence of an anomalous vascular pattern did not significantly influence the incidence rate of postoperative complications. In addition, no difference was evidenced between APV and MPV patients in terms of pathological findings, here including the rate of positive resection margins.

The potential correlation between an aberrant RHA and the onset of postoperative complications has been the focus of several studies. In particular, the accidental damage of aberrant vessels, as well as the alteration of local vascularization due to the excessive skeletonization of the aberrant arteries, has been proposed as the main predisposing features to postoperative complications, in particular BF. Indeed, as reported by Northover et al. [20], the proximal extra-hepatic biliary tree is vascularized by the RHA for almost the 40% of the total, while the remaining blood flow comes from the retroduodenal, the retroportal, and the gastroduodenal arteries. In this context, the presence of a RHA originating from the SMA and running along the posterior aspect of the retroportal lamina, that needs to be skeletonized in order to guarantee oncological radicality, may hypothetically increase the risk of bile duct (and subsequent hepaticojejunostomy) ischemia, potentially leading to a higher rate of BF. This issue has already been enlightened by Traverso in 1989 [5] and assessed by several authors in recent years. However, the majority of them did not evidence any statistical difference between MPV and APV patients. Indeed, Eshuis and Rammohan showed that, despite higher intraoperative technical difficulty, the presence of an rRHA or aRHA did not affect perioperative outcomes, including BF incidence [21, 22]. More recently, Alexakis conducted a matched analysis only on patients who underwent surgery for benign diseases, reporting similar rates of complications [23]. Our findings further corroborate these evidences since no differences were detected between APV and MPV patients both in terms of postoperative complications and, in particular, BF incidence (*p* = 0.2 and *p* = 0.58, respectively). This would underline how an adaptive system would take place in case of an aberrant RHA, maintaining a constant and efficient blood supply for the biliary duct stump vascularization.

Great concern has been also expressed about the potential role that an aberrant RHA may have on the oncological radicality, especially in terms of resection margins. For instance, an aRHA or rRHA generally arise from the SMA and run along the posterior aspect of the duodeno-cephalopancreatic block through the retroportal lamina. This last is widely recognized as the most frequent site of R1 and local tumor recurrence in case of infiltration or incomplete removal [24, 25]. In this scenario, the need of preservation of the anomalous artery, such as an aRHA and rHA, might hinder a proper oncological dissection, thus paving the way to a positive retropancreatic margin and to a dismal oncological result, increasing at the same time the risk of PPH due to the necessary skeletonization of the anomalous artery. Although the oncological role of vascular arterial resection is highly debated, some author [26] advise a vascular resection of the aberrant artery when the tumor is closer than 10 mm to the artery in order to achieve an R0 resection, eventually performing a vascular reconstruction through the interposition of the gastroduodenal stump [27, 28]. According to our findings, no difference has been evidenced between the MPV and APV cohorts in terms of resection margin positivity (here including the mesopancreas margin), in line with Turrini et al. that reported no difference in terms of margin status when the variant right hepatic artery was not directly infiltrated [9]. Moreover, no arterial vascular resections were needed intraoperatively, and PPH rate was similar between the two study cohorts. These outcomes may find justification in the importance of performing such complex procedure in high-volume referral centers [18, 29]. Specifically, the preoperative assessment of tumor resectability and vascular anatomy, performed in a multidisciplinary and specialized setting, has permitted to adequately select patients for PD, excluding those with evidence of tumor involvement of major vessels, notably candidate to neoadjuvant treatment. Similarly, the surgical expertise developed over the years has permitted to achieve similar PPH rate between the MPV and APV populations (*p* = 0.6), with even comparable operative time detected in two study cohorts (*p* = 0.79) and no accidental vascular resection during surgery.

This study presents several limitations especially due to its retrospective study design as well as to the relatively small sample size of the study cohort. On the counterpart, to our knowledge, this is the first study in the literature on this topic based on a PSM-derived cohort that has significantly limited the potential selection biases, making the two populations homogenous for the majority of confounding factors. Moreover, our findings derive from a high-volume tertiary referral center for the surgical treatment of pancreatic diseases, giving an objective and real picture of clinical outcomes when PDs with APV are performed in specialized centers.

In conclusion, the present analysis has confirmed that the presence of anomalies of the RHA does not significantly modify the postoperative clinical course nor the quality of surgical dissection. It is, however, implicit the need to perform this more complex procedure in high-volume centers and in a multidisciplinary setting. Nevertheless, additional studies with larger series are necessary to further corroborate our results.

## Data Availability

No datasets were generated or analysed during the current study.
